# Evolutionary conservation of VSX2 super-enhancer modules in retinal development

**DOI:** 10.1242/dev.202435

**Published:** 2024-07-12

**Authors:** Victoria Honnell, Shannon Sweeney, Jackie Norrie, Madison Parks, Cody Ramirez, Asha Jacob Jannu, Beisi Xu, Brett Teubner, Ah Young Lee, Claire Bell, Michael A. Dyer

**Affiliations:** ^1^ Department of Developmental Neurobiology at St. Jude Children's Research Hospital, Memphis, TN 38105, USA; ^2^ Center for Applied Bioinformatics at St. Jude Children's Research Hospital, Memphis, TN 38105, USA; ^3^ Wilmer Eye Institute, Johns Hopkins University School of Medicine, Baltimore, MD 21231, USA

**Keywords:** Retina, Super-enhancer, Organoid, Retinal development

## Abstract

Super-enhancers (SEs) are expansive regions of genomic DNA that regulate the expression of genes involved in cell identity and cell fate. We recently identified developmental stage- and cell type-specific modules within the murine *Vsx2* SE. Here, we show that the human *VSX2* SE modules have similar developmental stage- and cell type-specific activity in reporter gene assays. By inserting the human sequence of one *VSX2* SE module into a mouse with microphthalmia, eye size was rescued. To understand the function of these SE modules during human retinal development, we deleted individual modules in human embryonic stem cells and generated retinal organoids. Deleting one module results in small organoids, recapitulating the small-eyed phenotype of mice with microphthalmia, while deletion of the other module led to disruptions in bipolar neuron development. This prototypical SE serves as a model for understanding developmental stage- and cell type-specific effects of neurogenic transcription factors with complex expression patterns. Moreover, by elucidating the gene regulatory mechanisms, we can begin to examine how dysregulation of these mechanisms contributes to phenotypic diversity and disease.

## Teaser

**Summary:** Deleting individual super-enhancer modules in human embryonic stem cells can be used to study developmental stage- and cell type-specific effects of neurogenic transcription factors with complex expression patterns.

## INTRODUCTION

For proper retinal development to occur, thousands of genes must be turned on and turned off in a precise spatiotemporal order (Livesey and Cepko, 2001). Many genes encoding regulatory transcription factors are expressed in retinal progenitor cells during early stages of development and in a subset of differentiated cells at late stages of development (Castro et al., 2011; Haubst et al., 2004; Nishida et al., 2003). For example, *Sox2* is expressed in retinal progenitor cells and persists in differentiated Müller glia and starburst amacrine cells in the adult retina (Graham et al., 2003; Whitney et al., 2014). Likewise, *Pax6* is expressed in retinal progenitor cells and persists in amacrine cells, retinal ganglion cells and Müller glia (Collinson et al., 2003; Lalitha et al., 2020; Marquardt et al., 2001). Although these complex cell type-specific gene expression patterns have been well-characterized during retinogenesis, the underlying molecular mechanisms by which these genes are regulated is not well understood.

Previous studies have computationally identified hundreds of putative super-enhancers (SEs) across multiple stages of retinal development (Aldiri et al., 2017; Marchal et al., 2022). These expansive regions contain unusually high transcription factor binding, and are enriched for H3K27ac and H3K4me1 (Whyte et al., 2013). A major challenge in the field is correctly identifying which SEs regulate specific genes with complex developmental stage- and cell type-specific expression. This is challenging because SEs can regulate gene expression over a long distance (up to 1 Mb) and in either orientation.

We recently identified a SE upstream of the *Vsx2* gene that is necessary and sufficient for the complex expression pattern of *Vsx2* expression during development (Honnell et al., 2022). *Vsx2* is expressed in retinal progenitor cells and maintained in differentiated bipolar neurons and Müller glia (Buenaventura et al., 2018; Liu et al., 1994; Rowan and Cepko, 2004; Vitorino et al., 2009). This complex pattern of expression is achieved through distinct modules within the murine *Vsx2* SE (Honnell et al., 2022). One module (mR0-37) is responsible for expression in retinal progenitor cells and Müller glia. A second module (mR1-28) also contributes to retinal progenitor cell expression in concert with mR0-37. Deletion of mR0-37 leads to microphthalmia, and deletion of mR1-28 leads to a hypocellular retinal phenotype and loss of visual acuity but normal eye size (Honnell et al., 2022). The bipolar cell expression is regulated by a distinct module (mR3-17), and deletion of this module leads to a complete loss of bipolar neurons with no effect on retinal progenitor cell proliferation (Honnell et al., 2022). Taken together, these results suggest that the complex pattern of *Vsx2* expression during retinal development was achieved by evolution of a large SE with distinct modules that individually regulate expression at specific stages of development in particular cell types.

Mutations in the *Vsx2/VSX2* gene lead to microphthalmia in humans and mice due to a defect in retinal progenitor cell proliferation (Burmeister et al., 1996; Ferda Percin et al., 2000; Truslove, 1962). To determine whether the murine *Vsx2* SE modules are evolutionarily conserved in humans, we performed cross-species reporter gene assays. We also replaced mR0-37 with the orthologous sequence from the human *VSX2* SE (hR0-36) and showed functional rescue of microphthalmia. To study the role of individual *VSX2* SE modules in human retinal development, we deleted hR0-36 and hR3-16 in human embryonic stem cells (hESCs), and generated retinal organoids. For comparison, we also generated hESC lines with *VSX2* gene mutations. 3D retinal organoids derived from *hR0-36^Δ/Δ^* hESCs were smaller and made up primarily of Müller glia, consistent with the phenotype in *mR0-37^Δ/Δ^* mice (Honnell et al., 2022). There was also a defect in bipolar cell development in the 3D retinal organoids derived from *hR3-16^Δ/Δ^* hESCs, but it was bipolar subset specific. These data highlight the importance of *in vivo* validation of SE function across species and of dissection of individual modules that contribute to the complex patterns of gene expression during development. These data may also be used to identify genomic regions where non-coding genetic lesions contribute to defects in eye development and function.

## RESULTS

### The *Vsx2/VSX2* SE enhancer modules are evolutionarily conserved

**Fig. 1.**
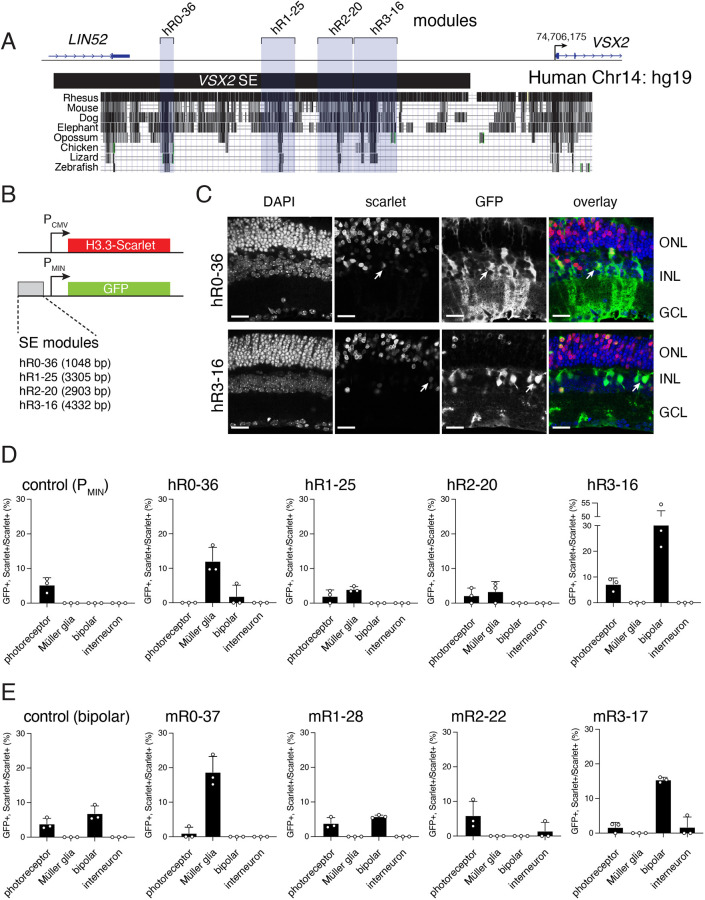
**The VSX2 SE contains evolutionarily conserved modules that are sufficient for driving reporter expression between species.** (A) Map of the evolutionarily conserved modules in the human *VSX2* SE (blue shading). (B) Plasmids used for reporter assays for *in vivo* square wave electroporation with coordinates of each SE module (hg19 build). A minimal promoter (P_MIN_) is upstream of GFP, and a strong constitutive promoter (P_CMV_) is used for the nuclear H3.3-Scarlet reporter. (C) Micrographs of GFP (green) and Scarlet (red) expression at P21 from square wave electroporation of P0 mice. Arrows indicate Müller glia for the hR0-36 plasmid and bipolar neurons for the hR3-16 fragment. (D,E) Quantification of reporter-positive retinal neurons for three biological replicates for each human reporter construct. The negative control (D) is the empty P_MIN_ plasmid that has only low level background expression in rods. The positive control (E) has a previously identified bipolar-specific element. Data are mean±s.d. ONL, outer nuclear layer; INL, inner nuclear layer; GCL, ganglion cell layer. Scale bar: 25 µm.

Using integrated epigenetic analyses, we previously identified a 41 kb SE upstream of the *Vsx2* gene in mice (Fig. S1) (Norrie et al., 2019). Within the *Vsx2/VSX2* SE are four evolutionarily conserved modules: mR0-37/hR0-36, mR1-28/hR1-25, mR2-22/hR2-20 and mR3-17/hR3-16. The nomenclature for the modules indicates the species (murine, m; human, h), module number (R0-3) and distance from the start of transcription of the target gene (16-37 kb) (Honnell et al., 2022) (Fig. 1A, Fig. S1). To determine whether these human modules are sufficient for driving expression during murine retinal development, we subcloned them upstream of a minimal promoter (P_MIN_) followed by a GFP reporter gene (Kim et al., 2008) (Fig. 1B). Plasmids were square wave electroporated in the mouse retina *in vivo* at P0 with a constitutive H3.3 Scarlet reporter plasmid (Fig. 1B). Three weeks later, retinae were harvested, fixed in paraformaldehyde, vibratome sectioned and imaged on a confocal microscope (Fig. 1C). We scored the proportion of GFP^+^, Scarlet^+^/Scarlet^+^ cells for each cell type for each reporter plasmid. hR0-36 was sufficient for expression in Müller glia and hR3-16 was sufficient for expression in bipolar neurons (Fig. 1D). The activity of the human *VSX2* SE modules was similar to the orthologous regions in the murine *Vsx2* SE (Fig. 1E). In our previous study, we showed that mR0-37 was active in retinal progenitor cells in E14.5 retinal explants and Müller glia, and that mR3-17 was active in bipolar neurons (Honnell et al., 2022). The evolutionarily conserved sequences of the *Vsx2* SE that overlapped with the *Lin52* gene did not have activity in our reporter assay (Fig. S1). We further refined the active modules (1-5 kb) into discrete elements (300-500 bp) based on evolutionary conservation (Fig. S1). The mR3-17d element overlaps the minimal enhancer sequence previously found by the Cepko lab to be sufficient for bipolar cell expression (Kim et al., 2008). We also combined the functional modules (R0+R1+R3) and elements (mini enhancer), and showed that they recapitulate the activity of the full 41 kb SE in our reporter gene assay (Fig. S1).

### hR0-36 can functionally replace mR0-37 in the mouse retina *in vivo*

**Fig. 2.**
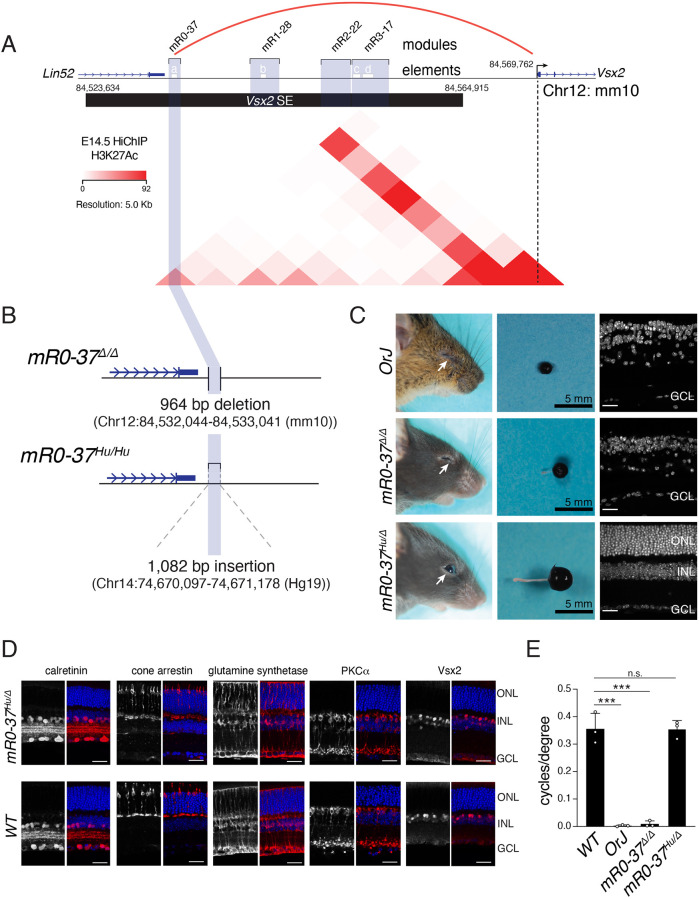
**The hR0-36 *VSX2* SE module can functionally replace the mR0-37 *Vsx2* SE module *in vivo*.** (A) The evolutionarily conserved modules (blue shading) and individual elements within the modules (a-d, white bars) for the murine *Vsx2* SE. H3K27Ac HiChIP is shown for E14.5 retinae, indicating that mR0-37 is in close proximity to the *Vsx2* promoter by looping out the intervening chromatin. (B) The coordinates of the deletion of mR0-37 and replacement of the murine sequence with the corresponding human sequence from hR0-36. (C) Photographs of mice, eyes and DAPI-stained retinal sections for the eyes of *Vsx2* gene knockout mice (*OrJ*), *mR0-37^Δ/Δ^* and *mR0-37^Hu/Δ^* mice. (D) Representative micrographs of immunostaining of vibratome sections of wild-type and *mR0-37^Hu/Δ^* adult retinae. A subset of amacrine cells (calretinin), cones (cone arrestin), Müller glia (glutamine synthetase) and bipolar neurons (PKCα, Vsx2) is shown. (F) Photopic vision (cycles/degree) in *Vsx2* gene knockout mice (*OrJ*), *mR0-37^Δ/Δ^* and *mR0-37^Hu/Δ^* mice (unpaired two-tailed *t*-test; ***P≤0.0005; n.s., not significant). Data are mean±s.d. *n*=3 biologically independent mice for each mouse strain. Scale bars: 25 µm.

H3K27Ac HiChIP on E14.5 retinae predicts that mR0-37 interacts with the *Vsx2* promoter by looping out the intervening sequence (Fig. 2A). To determine whether the hR0-36 module of the *VSX2* SE can functionally replace mR0-37 in retinal development, we used CRISPR-Cas9 to replace the 964 bp of mR0-37 with the 1082 bp orthologous human sequence hR0-36 in mice (Fig. 2B). The *mR0-37^Δ/Δ^* mice have microphthalmia, and insertion of a single copy of hR0-36 completely rescues the phenotype in *mR0-37^Hu/Δ^* mice (Fig. 2C). Eye size, retinal thickness and organization, and visual acuity were indistinguishable from wild-type C57Bl/6 mice (Fig. 2C-E). These data demonstrate that hR0-36 can functionally replace mR0-37 in retinal development *in vivo*.

Mutations in the *Vsx2* gene and deletion of the mR0-37 *Vsx2* SE module leads to microphthalmia due to defects in retinal progenitor cell proliferation (Burmeister et al., 1996; Ferda Percin et al., 2000; Honnell et al., 2022; Livne-Bar et al., 2006; Truslove, 1962). The small eyes in the *Vsx2* gene or SE knockouts resemble those of subterranean mammals, such as the shrew and mole, but the eye is not completely lost as it is in blind cave fish (Partha et al., 2017). Indeed, the progenitor/Müller glial cell module (mR0-37/hR0-36) has more sequence divergence in those species than the other *Vsx2/VSX2* SE modules. This may suggest that changes in the *Vsx2/VSX2* SE sequence contribute to eye size and that there may be some evolutionary advantage to retaining a rudimentary eye that is capable of sensing light but has no visual acuity. To determine whether there is any light detection in the microphthalmic eyes of the *Vsx2/VSX2* gene and SE mutants, we tested their photoentrainment using running-wheel activity. The rod and cone circuits can contribute to photoentrainment as well as intrinsically photosensitive retinal ganglion cells that express melanopsin (*Opn4*) (Hattar et al., 2002). For controls, we used *Rd1^–/–^* mice that lack rods and cones, *Opn4^–/–^* mice that lack melanopsin and the double knockouts (*Rd1^–/–^;Opn4^–/–^*). As expected, the *Rd1^–/–^* mice can photoentrain through the melanopsin pathway and the *Opn4^–/–^* mice can photoentrain through the rod/cone pathway (Fig. S2). The double knockout mice cannot photoentrain (Fig. S2). The *OrJ* and *mR0-37*^Δ*/*Δ^ mice do not photoentrain, suggesting that they have defects in both the rod/cone pathway and melanopsin pathways (Fig. S2).

### hR0-36 is required for retinal progenitor cell proliferation in 3D retinal organoids

**Fig. 3.**
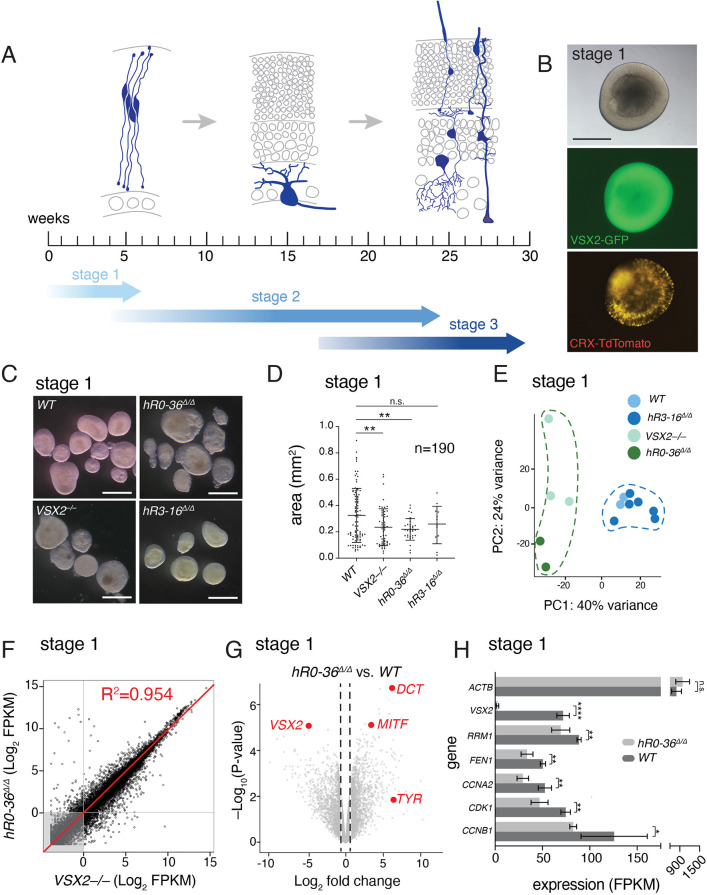
***hR0-36^Δ/Δ^* retinal organoids are smaller and resemble *VSX2^–/–^* retinal organoids.** (A) The staging of retinal organoids (Capowski et al., 2019). Stage 1 organoids contain retinal progenitor cells (blue) and some newly postmitotic cells at the basal surface (gray). They also start to accumulate newly postmitotic cones at the later stages of stage 1 (Table S1). By stage 2, most cells have exited the cell cycle (gray) and retinal ganglion cells (blue) are starting to differentiate. At stage 3, retinal ganglion cells may be lost, and the other major classes of retinal cell types differentiate (blue). (B) Micrographs of live retinal organoid at stage 1 showing the dual reporter with green retinal progenitor cells (*VSX2-GFP*) and red photoreceptors (*CRX-TdTomato*). (C) Micrographs of representative stage 1 organoids for each genotype. (D) Scatter dot plot of organoid area in mm^2^. Each dot represents a single retinal organoid measurement. *VSX2*^–/–^ and *hR0-36^Δ/Δ^* retinal organoids are significantly smaller than wild-type retinal organoids (unpaired two-tailed *t*-test, **P<0.01). There is no significant difference observed between R3^Δ/Δ^ and wild-type retinal organoid area (unpaired two-tailed *t*-test, *P*=0.7811). (E) Principal component analysis plot of bulk RNA-seq from stage 1 retinal organoids. Each dot represents a distinct biological replicate. (F) Scatterplot of Log2-FPKM from bulk RNA-seq for *VSX2^–/–^* and *hR0-36^Δ/Δ^* retinal organoids. Each dot is an individual gene; the red line is the linear regression with a correlation coefficient of 0.954. (G) Volcano plot of *hR0-36^Δ/Δ^* retinal organoids versus wild type with some highlighted upregulated (*DCT*, *MITF* and *TYR*) and downregulated (*VSX2*) genes. (H) FPKM values for representative genes in *hR0-36^Δ/Δ^* and wild-type retinal organoids (unpaired two-tailed *t*-test; *P<0.05, **P<0.01, ****P<0.0001; n.s., not significant). Data are mean±s.d. FPKM, fragments per kilobase per million reads. Scale bars: 0.5 mm.

To understand the effect of the *Vsx2* SE in a model of human retinal development, we generated human retinal organoids by following an established protocol with three defined developmental stages (Capowski et al., 2019) (Fig. 3A). Using CRISPR-Cas9, we deleted hR0-36 (*hR0-36*^Δ*/*Δ^), hR3-16 (*hR3-16*^Δ*/*Δ^) and generated frameshift mutations in exon 2 (*VSX2^–/–^*) in H9 human embryonic stem cells (hESCs). The parent H9 hESC line contained a dual reporter that labels cells expressing *VSX2* (GFP) and *CRX* (TdTomato), so that we can readily monitor retinal development in live cultures (Fig. 3B). Two independent sublines were created and analyzed for each deletion. No differences in sublines were observed by RNA seq, qRT-PCR or morphological analysis by immunohistochemistry (Table S1).

At stage 1, all retinal organoid lines express *CRX-TdTomato*, which marks photoreceptors. Wild-type and *hR3-16*^Δ*/*Δ^ retinal organoids expressed *VSX2-GFP*, which marks retinal progenitor cells. GFP was not visible by fluorescent light microscope in *VSX2^–/–^* and *hR0-36*^Δ*/*Δ^ retinal organoids. At this stage, the size of the *VSX2^–/–^* and *hR0-36*^Δ*/*Δ^ retinal organoids was slightly smaller but was not statistically significant (Fig. 3C,D). However, bulk RNA-seq showed that the *VSX2^–/–^* and *hR0-36*^Δ*/*Δ^ retinal organoids were the same as each other and distinct from wild-type and *hR3-16*^Δ*/*Δ^ retinal organoids (Fig. 3E-H and Table S1). At stage 1, *hR0-36*^Δ*/*Δ^ retinal organoids downregulate *VSX2* and upregulate microphthalmia-associated transcription factor (*MITF*) (Bharti et al., 2008; Horsford et al., 2005; Rowan et al., 2004; Zou and Levine, 2012) (Fig. 3G). These data are consistent with an earlier study showing that human retinal organoids derived from a patient with *VSX2*-mediated microphthalmia also exhibit upregulation in *MITF* compared with a non-affected sibling (Phillips et al., 2014). Additional upregulated genes include targets of *MITF*, namely *DCT* and *TYR* (Kawakami and Fisher, 2017) (Fig. 3G). In addition to *VSX2*, several genes important for retinal progenitor cell proliferation were significantly downregulated in the *hR0-36*^Δ*/*Δ^ retinal organoids relative to wild type (Fig. 3H).

**Fig. 4.**
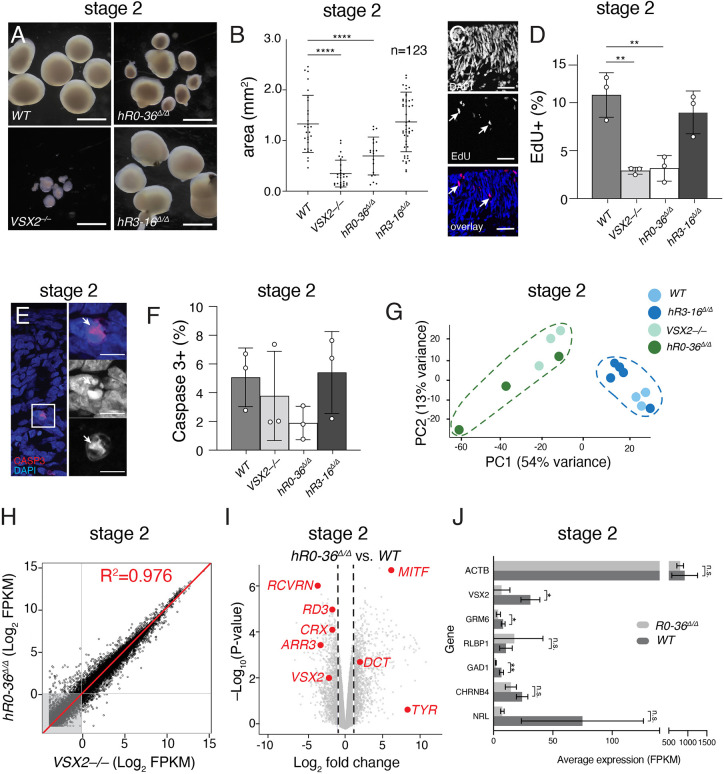
***hR0-36^Δ/Δ^* retinal organoids have defects in proliferation and neuronal differentiation**. (A) Micrographs of stage 2 retinal organoids for each genotype. The heterogeneity of *hR0-36^Δ/Δ^* retinal organoids are apparent at this stage. (B) Scatter dot plot of organoid area in mm^2^. Each dot represents a single retinal organoid measurement. *VSX2*^–/–^ and *hR0-36^Δ/Δ^* retinal organoids are significantly smaller than wild-type retinal organoids (unpaired two-tailed *t*-test, ****P<0.0001). (C) Representative micrographs of wild-type retinal organoid at stage 2 showing EdU (red) and nuclear stain (blue). Arrows indicate representative EdU-positive cells. (D) EdU scoring at stage 2 for each genotype (**P<0.01). Data are mean±s.d. (E) Representative micrograph of stage 2 retinal organoids stained for activated caspase 3 (red) as a marker of cell death (arrows). Nuclei are stained with DAPI (blue). (F) Percentage of activated caspase 3-positive nuclei. There was no significant difference across genotypes. Data are mean±s.d. (G) Principal component analysis plot of bulk RNA-seq from stage 2 retinal organoids. Each dot represents a distinct biological replicate. (H) Scatterplot of Log2-FPKM from bulk RNA-seq for *VSX2^–/–^* and *hR0-36^Δ/Δ^* retinal organoids. Each dot is an individual gene; the red line is the linear regression with a correlation coefficient of 0.976. (I) Volcano plot of *hR0-36^Δ/Δ^* retinal organoids versus wild type with some highlighted upregulated (*DCT*, *MITF* and *TYR*) and downregulated (*VSX2*, *ARR3*, *CRX*, *RD3* and *RCVRN*) genes. (J) FPKM values for representative genes in *hR0-36^Δ/Δ^* and wild-type retinal organoids (unpaired two-tailed *t*-test; *P<0.05, **P<0.01; n.s., not significant). Data are mean±s.d. FPKM, fragments per kilobase per million reads. Scale bars: 0.5 mm in A; 25 µm in C; 5 µm in E.

By the end of stage 2, most retinal progenitor cells are expected to have fully differentiated, and all neuronal cell types are present (see Fig. 3A) (Capowski et al., 2019). At this stage, the *VSX2^–/–^* and *hR0-36*^Δ*/*Δ^ retinal organoids were significantly smaller compared with wild-type retinal organoids (Fig. 4A,B). To measure the proportion of cells in S phase, we labeled stage 2 (day 90) retinal organoids for 1 h with EdU immediately before harvest. Organoids were fixed in paraformaldehyde, cryoembedded, cryosectioned and stained for EdU and nuclei (DAPI). Samples were de-identified and confocal images were scored for the proportion of EdU^+^ nuclei in three independent samples for each genotype (Fig. 4C,D). There was a significant reduction in the proportion of EdU^+^ cells in the *VSX2^–/–^* and *hR0-36*^Δ*/*Δ^ retinal organoids compared with wild-type retinal organoids, consistent with their smaller size (Fig. 4D). We also analyzed cell death by immunostaining for activated caspase3 (Fig. 4E). There was no significant difference in the proportion of Caspase3^+^ cells across all samples (Fig. 4F). The bulk RNA-seq analysis was similar to stage 1 with *VSX2^–/–^* and *hR0-36*^Δ*/*Δ^ retinal organoids clustering separately from the wild type and *hR3-16*^Δ*/*Δ^ retinal organoids (Fig. 4G). The *VSX2^–/–^* and *hR0-36*^Δ*/*Δ^ retinal organoids were highly correlated with each other (Fig. 4H) and had defects in the differentiation of retinal cell types such as photoreceptors (Fig. 4I,J). Approximately half of the *hR0-36*^Δ*/*Δ^ retinal organoids were similar in size to wild type and half were significantly smaller than wild type (see Fig. 4A), and this heterogeneity was reflected in the PCA plot for the bulk RNA-seq (Fig. 4G).

**Fig. 5.**
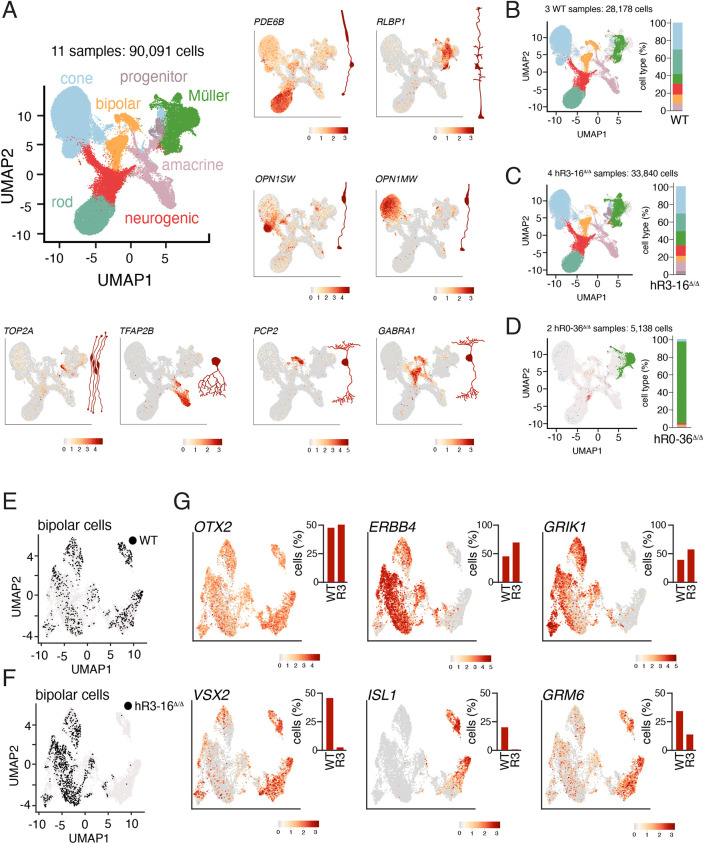
***hR3-16^Δ/Δ^* retinal organoids have defects in bipolar neurons.** (A) UMAP of scRNA-seq for stage 3 human retinal organoids, including three wild-type, four *hR3-16^Δ/Δ^* and four *hR0-36^Δ/Δ^* retinal organoids. For the *hR0-36^Δ/Δ^* retinal organoids, we used two large and two small retinal organoids. Representative feature plots for genes enriched in individual cell types are shown for rods (*PDE6B*), Müller glia (*RLBP1*), cones (*OPN1SW* and *OPN1MW*), retinal progenitor cells (*TOP2A*), amacrine cells (*TFAP2B*) and bipolar neurons (*PCP2* and *GABRA1*). (B-D) UMAP plots of cell types for three wild-type (B), four *hR3-16^Δ/Δ^* (C) and two small *hR0-36^Δ/Δ^* (D) retinal organoid scRNA-seq data. The large *hR0-36^Δ/Δ^* retinal organoids were indistinguishable from wild type. Stack bar plots show the quantitation of cell types. (E,F) UMAP plots of bipolar neurons from the data shown in A. In the wild-type retinal organoids, cells are distributed across the clusters (E), but they are enriched in the OFF cone bipolar cluster in the *hR3-16^Δ/Δ^* retinal organoids (F). (G) Representative feature plots of expression of pan-bipolar genes (*OTX2* and *VSX2*), OFF cone bipolar cells (*ERBB4* and *GRIK1*) and rod bipolar/ON cone bipolar neurons (*ISL1* and *GRM6*). Quantitation is shown for wild-type and *hR3-16^Δ/Δ^* (R3) organoids for each feature plot.

To elucidate the cellular heterogeneity in organoids of different size, we performed scRNA-seq of two biological replicates for small *hR0-36*^Δ*/*Δ^ retinal organoids, four biological replicates for *hR3-16*^Δ*/*Δ^ retinal organoids, two biological replicates for large *hR0-36*^Δ*/*Δ^ retinal organoids and three biological replicates for wild-type retinal organoids at stage 3 (Fig. 5A-D). The small *hR0-36*^Δ*/*Δ^ retinal organoids were primarily made up of Müller glia, as seen in the mR0-37^Δ/Δ^ mice (Fig. 5D). The large *hR0-36*^Δ*/*Δ^ retinal organoids were more like wild-type retinal organoids. All scRNA-seq data are freely available for visualization in the R Shiny app at (https://proteinpaint.stjude.org/iRNDb_v2/). This heterogeneity highlights the importance of performing biological replicates, culturing individual retinal organoids separately and the variability that can occur even with identical conditions. A similar distribution was observed with two independent sublines lacking hR0-36 (data not shown). There were more subtle shifts in cell type distribution in the *hR3-16*^Δ*/*Δ^ retinal organoids, with a decrease in rods from 27.8% to 19.2%, a decrease in bipolar neurons from 9.7% to 6.2% and an increase in Müller glia from 11.5% to 16.1% (Fig. 5A-C). Taken together, our data suggest that the hR0-36 module is necessary for retinal progenitor cell proliferation and that the *hR0-36*^Δ*/*Δ^ human retinal organoids recapitulate the small-eye phenotype observed in mice with microphthalmia. Moreover, the hR3-16 module may have a more-subtle defect in bipolar neuron development in human retinal development than in the mouse.

### hR3-16 is required for bipolar cell subtype specification

As described above, the *hR3-16*^Δ*/*Δ^ retinal organoids were like wild-type retinal organoids in size and gene expression from bulk RNA-seq. To determine whether there was any defect in bipolar neuron development, we re-clustered the bipolar neurons and analyzed the expression or bipolar subtype specific genes. Single cell RNA-seq of murine retinal bipolar neurons has identified at least 15 subtypes of bipolar neurons that broadly fall into the categories of ON cone bipolar, OFF cone bipolar or rod bipolar (RB) (Shekhar et al., 2016). Rather than a complete loss of bipolar cells, as in the retinae of *hR3-17*^Δ*/*Δ^ mice, there was a reduction of RB and ON cone bipolar neurons, and an increase in OFF cone bipolar neurons (Fig. 5E-G). For example, *GRM6* and *ISL1* are expressed in RB and in several subtypes of ON cone bipolar neurons in the murine retina (Shekhar et al., 2016). The proportion of bipolar neurons expressing *GRM6* and *ISL1* was reduced in the *hR3-16*^Δ*/*Δ^ retinal organoids relative to wild type (Fig. 5G). There was a compensatory increase in the proportion of OFF bipolar neurons expressing genes such as *ERBB4* and *GRIK1* (Fig. 5E-G).

## DISCUSSION

Super-enhancers have been found to be important for normal developmental processes, and have been implicated as drivers of disease due to enrichment of disease-associated genetic variation (Hnisz et al., 2013). There is growing evidence that many super-enhancers important for development in vertebrates are evolutionarily conserved (Khan et al., 2018; Pérez-Rico et al., 2017; Zhang et al., 2022). Here, we demonstrate that the *Vsx2/VSX2* super-enhancer has distinct evolutionarily conserved modules that have similar function in humans and mice. One of the modules (hR0-36) important for expression of *VSX2* in retinal progenitor cells and Müller glial can functionally replace the orthologous module (mR0-37) in mouse retinal development. The module that is necessary and sufficient for bipolar cell development in the murine retina (mR3-17) is important for bipolar neuron subtype specification in human retinal organoids (hR3-16). Our results emphasize the importance of performing *in vivo* validation of SEs and their individual functional modules across species.

### Identification of *Vsx2/VSX2* SE modules

Four murine *Vsx2* SE modules (mR0-37, mR1-28, mR2-22 and mR3-17) were initially identified by integrating our ChIP-seq data and evolutionary conservation (Kent et al., 2002; Raney et al., 2014). Reporter assays further refined the functional modules in the human and murine *Vsx2/VSX2* SE. Specifically, mR0-37/hR0-36 and mR3-17/hR3-16 had developmental stage- and cell type-specific activity. Indeed, reporter assays were useful for narrowing down the functional element of mR3-17 to only 164 bp. This is useful for identifying transcription factor-binding sites and searching for genomic variants that may alter *VSX2* expression in human with retinopathies. Although the combination of ChIP-seq and evolutionary conservation are useful for identifying candidate modules of developmental SEs, *in vivo* functional studies are essential. For example, the mR2-22/hR2-21 module had no activity in reporter assays, despite being the most evolutionarily conserved module within the SE. *In vivo* knockout of mR2-22 had no effect on retinal development (Honnell et al., 2022), indicating that it is neither necessary nor sufficient for expression of *Vsx2* in retinal development. Similarly, the mR1-28/hR1-25 module did not have any significant activity in reporter assays but knockout of mR1-28 in mice led to a hypocellular retina and loss of visual acuity (Honnell et al., 2022). Therefore, *in vivo* functional validation by knockout is required to determine the function of each SE module.

### *Vsx2/VSX2* SE and control of eye size

SEs are implicated in driving normal development genetic lesions in SEs can contribute to human disease (Hnisz et al., 2013; Parker et al., 2013; Whyte et al., 2013). However, the contribution of developmental SEs to phenotypic diversity among species is still being explored. A previous study by the Clark Lab examined the evolutionary rate of subterranean mammalian enhancers implicated in eye development (Partha et al., 2017). This was guided by the premise that, despite being distantly related, many subterranean mammals have poor eyesight, thus unique pressures of the underground environment may promote convergent evolution. They identified 17 eye-specific enhancers in the mole that diverged at significantly accelerated rates relative to other enhancers believed to be important for eye development. One of those 17 enhancers was the retinal progenitor cell module (mR0-37/hR0-36) within *Vsx2/VSX2* SE.

Unlike blind cave fish, the subterranean species retain a small eye and optic nerve, similar to *OrJ* and *mR0-37^Δ/Δ^* mice. It is possible that there is some advantage in the ability to sense light, even if they lack visual acuity. To test this, we assessed photoentrainment of our microphthalmic mice (*OrJ* and *mR0-37^Δ/Δ^*) using running wheels to measure activity and circadian rhythm. Photoentrainment can be achieved by the photoreceptor circuit or from intrinsically photosensitive retinal ganglion cells that express melanopsin (*Opn4*). Mice lacking photoreceptors (*Rd1*) and melanopsin (*Opn4*) fail to photoentrain (Hattar et al., 2002). Similarly, mice lacking mR3-17 and *Opn4* fail to photoentrain because the light input from the photoreceptors cannot be transmitted to the retinal ganglion cells due to the complete absence of bipolar neurons. The *OrJ* and *mR0-37^Δ/Δ^* mice do not photoentrain, suggesting that they are not transmitting any light input via the photoreceptor pathway or the melanopsin pathway.

### Human *VSX2* SE function

3D human retinal organoids serve as a powerful tool for modeling human retinal development and disease (Foltz and Clegg, 2019; Kruczek and Swaroop, 2020; Ludwig et al., 2023; Phillips et al., 2014; Saha et al., 2022; Wahle et al., 2023). To understand the role of the *VSX2* SE modules in human retinal organoids, we individually deleted hR0-36 and hR3-16, and assessed their growth and development over three stages of development (Capowski et al., 2019). The *hR0-36^Δ/Δ^* exhibited heterogeneity in size and gene expression. Half of the organoids were similar in size to the wild-type retinal organoids, and had bulk and single-cell RNA-seq profiles that were indistinguishable from wild type*.* The small *hR0-36^Δ/Δ^* retinal organoids were primarily made up of Müller glia and resembled the *VSX2^–/–^* retinal organoids. The cell type distribution of the small *hR0-36^Δ/Δ^* retinal organoids was similar to that of the retinae from *mR0-37^Δ/Δ^* mice (Honnell et al., 2022). The heterogeneity was observed with two independent *hR0-36^Δ/Δ^* hESC sublines, so it is unlikely to be an artifact of the CRISPR-Cas9 targeting. Taken together, these data suggest that mR0-37 and hR0-36 are evolutionarily conserved and contribute to *Vsx2/*VSX2 expression in retinal progenitor cells.

Deletion of mR3-17 in mice led to a complete loss of bipolar neurons. In contrast, deletion of hR3-16 in retinal organoids led to a reduction of RB and ON cone bipolar neurons. It is important to emphasize that our analysis relied exclusively on molecular markers of bipolar subtypes, and we could not confirm differences in morphology because lamination in retinal organoids is highly variable. Therefore, it is possible that changes in VSX2 expression because of the SE deletion simply change the gene expression program and do not actually alter the distribution of bipolar subtypes. Indeed, *ISL1* was completely absent from the bipolar neurons in our scRNA-seq analysis, suggesting that this may be a direct target of VSX2. It is also possible that there are additional modules or elements in the human *VSX2* SE that control bipolar cell development or that VSX2 is not required for specification of all bipolar cell types in humans. In either case, combining in-depth chromatin profiling with sequence conservation across species, reporter assays and *in vivo* deletion experiments provides new insights into functional modules of developmental SEs and their conservation across species. This is important for identifying genomic regions that may be altered in individuals with microphthalmia or other retinopathies.

## MATERIALS AND METHODS

### Mouse strains

All animal procedures and protocols were approved by the St. Jude Laboratory Animal Care and Use Committee under protocol number 393-100500. All studies conform to federal and local regulatory standards. Mice were housed on ventilated racks on a standard 12 h light-dark cycle. Wild-type C57BL/6J mice were purchased from the Jackson Laboratory (000664). For timed pregnancy, individual male mice were housed with three females in a single cage. Plugged/pregnant females (identified by visual examination) were isolated, and embryos or pups were harvested at the appropriate time. Both males and females were combined for this study.

The coordinates of the Hg19 human regions were determined by entering the mouse coordinates and then using the ‘LiftOver’ tool on the UCSC genome browser. The mR0-37^Hu^ mice were created using CRISPR-Cas9. Briefly, ten 3- to 4-week-old C57BL/6J female mice from Jackson Labs were superovulated with 5 units of gonadotrophin from pregnant mare's serum (PMSG from ProSpec) and, 48 h later, with 5 units of human chorionic gonadotrophin (hCG from Sigma). After overnight mating with *mR0-37^Δ/Δ^* males, the females were euthanized and oocytes were harvested from the ampullae. The protective cumulus cells were removed using hyaluronidase, and the oocytes were washed and graded for fertilization by observing the presence of two pronuclei. A mixture of 10 ng/μl sgRNA (IDT), 30 ng/μl SpCas9 protein (St. Jude Protein Production Core) and 5 ng/μl ssDNA (Azenta Life Sciences) was injected into the pronucleus of oocytes. The injected oocytes were then returned to culture media (M16 from Millipore or A-KSOM from Millipore) and later the same day transferred to day 0.5 pseudopregnant fosters (7- to 10-week-old CD-1 females from Charles River Laboratories mated to vasectomized CD-1 males). Pups were born after 19 days gestation and sampled at day 7-10 for genotyping. Animals positive for the insertion were weaned at day 21 and at 6 weeks of age, and were backcrossed to *mR0-37^Δ/Δ^* mice. The gRNA for insertion was 5′-GCAGGCCATGTGCTCGTCTC-3′. The primers used to analyze the 5′ junction of the insertion were: 5′-CACATACCGTCCGCCTCACACCTCA-3′ and 5′-CCAAATGCCAAAAAGCCTAC-3′. The internal human specific primers were: 5′-GCTGTGGGCAGAGAGGTAAG-3′ and 5′-GCAGAATTCACTCACGTGCT-3′. The primers used to analyze the 3′ junction were 5′-TGGTAGCATCACTCCAGGAA-3′ and 5′-ACCAGCCAACCTTCTGAGTCACACA-3′.

### Photoentrainment

Mice were individually housed with one wireless running wheel (Med Associates, ENV-047). Lights were on a 12 h light/dark schedule throughout the 36 day study. The room was light tight, and welfare checks/cage changes were quietly carried out during light-on periods. For the first 17 days, the lights went off at noon and turned on at midnight. On day 18, the light schedule shifted by forward by 6 h and the lights turned off at 6 PM and on at 6 AM. WheelMan software collected wheel rotations and was used for Actogram and Period Length analyses.

### Plasmid constructs

All plasmids were generated by GenScript and are deposited in Addgene (see supplementary Materials and Methods for further details).

### Immunostaining

Mouse retinae were fixed in 4% paraformaldehyde overnight at 4°C and washed three times in PBS. They were then embedded in 4% low melting point agarose and vibratome sectioned at 50 µm. Calretinin (Chemicon, MAB1568) was used at 1:100; cone arrestin (Millipore AB15282) was used at 1:5000; glutamine synthetase (BD Pharmingen 610518) was used at 1:100; PKCα (Upstate 05-154) was used at 1:5000; and Vsx2 (Exalpha X1180P) was used at 1:200. Blocking was performed in PBS with 5% matched normal serum based on the species of the secondary antibody with 0.5% Triton X-100. We used donkey anti-mouse (Vector Labs BA2000), goat anti-rabbit (Vector Labs BA-1000) and rabbit anti-sheep (Vector Labs BA-6000). After secondary antibody incubation (2 h), they were washed twice with PBS and incubated with ABC reagent (Vector Laboratories, PK6100) for 30 min. We then used tyramide Cy3 (PerkinElmer, FP1046) for 10 min at room temperature and washed twice in PBS followed by DAPI at 1:1000 in PBS. Slices were mounted in Prolong gold reagent and imaged on a Zeiss LSM700 confocal microscope.

### hESC lines

All studies conform to federal and local regulatory standards. The double reporter parent cell line (H9 VSX2-GFP/CRX-TdTomato) was a gift from the laboratories of Drs David Gamm and Don Zack. The line was validated by powerplex and tested for mycoplasma. The hR0-36^Δ/Δ^ and hR3-16^Δ/Δ^ lines were generated using CRISPR-Cas9. H9 *VSX2*-GFP/*CRX*-TdTomato hESCs were pretreated for 1 h in StemFlex (Thermo Fisher Scientific) supplemented with 1× RevitaCell (Thermo Fisher Scientific). After pretreatment, ∼1×10^6^ cells were nucleofected (Lonza, 4D-Nucleofector X-unit) with precomplexed ribonuclear proteins (RNPs) consisting of 120 pmol of chemically modified sgRNA (Synthego), 40 pmol of Cas9 protein (St. Jude Protein Production Core) and 1.5 μg of ssODN donor in a small (20 μl) cuvette using solution P3 and program CB-150 according to the manufacturer's recommended protocol. Single cells were distributed to wells of a 96-well dish 5 days after transfection in prewarmed (37°C) StemFlex media supplemented with 1X CloneR (Stem Cell Technologies) into Vitronectin XF (Stem Cell Technologies) coated 96-well plates. Clones were screened for the desired modification via gel electrophoresis and targeted deep sequencing on a Miseq Illumina sequencer, as previously described (Narina et al., 2023). NGS analysis of clones was performed using CRIS.py (Connelly and Pruett-Miller, 2019). Correctly modified clones were identified, expanded and their sequence confirmed. Cell identity was authenticated using the PowerPlex Fusion System (Promega) performed at the Hartwell Center (St. Jude, Memphis, TN, USA) and tested negative for mycoplasma with the MycoAlertTMPlus Mycoplasma Detection Kit (Lonza). The hR0-36 gRNAs were: 5′-GGUUGUAAGUUCUGAUGGAC-3′ and 5′-UGUGUUAGCUUUCUUUGCAA-3′. The hR3-16 gRNAs were: 5′-CAUGAGCUGUGAGCCUCUUC-3′ and 5′-UCUAGGAAGUUUUCCCUGCA. The PCR primers for hR0-36 were 5′-GCCAAGGTGGATGGTCGCAGAAGCC-3′ and 5′-AGGCAGGGGTTGGGGACACAAT-3′. The PCR primers for hR3-16 were 5′- CTGTTGATGGTGCTGGCTGGACAGT-3′ and 5′-GCAGCGTGGTGTAGTGGTTA-3′.

### Retinal organoids

Retinal organoids were grown and staged according to a previous publication (Capowski et al., 2019). Each organoid was placed in one well of a 24-well plate (1 organoid/well) with 1 ml of 3D Retinal Differentiation Media containing 1:1000 EC23 (3D RDM) and incubated at 37°C. Media changes occurred weekly and mycoplasma testing occurred every 2 months. After 100 days, 3D RDM media changes did not include EC23. Human retinal organoids were individually imaged at each stage using at 1.6× objective. For retinal organoid size quantitation from the images, we used 212 images to train nnUnet, a convolutional neural network, to segment the organoids and quantify the area of the segment using scikit-image library (Isensee et al., 2021; van der Walt et al., 2014). The tracing of each organoid was manually reviewed to ensure accuracy. For scRNA-seq, retinal organoids were dissociated using the Worthington papain kit by adding 40 U papain (Worthington, LS003119) to 400 μl of papain buffer and incubating at 37°C for 15 min. Organoids were individually submerged in retinal explant media (REM) and placed on ice. 400 μl of papain buffer was added to each retina and incubated at 37°C. Tubes were agitated twice at 5 min intervals and 40 μl of DNAse solution (DS) was added and incubated at 37°C for an additional 5 min. The cell suspension was filtered through a 40 μm cell strainer (Falcon, 352340) and the filter was washed with PBS to bring the total volume to 1.4 ml.

### RNA isolation

RNA was extracted from individual TRIzol (Life Technologies) preparations using a phenol-chloroform extraction. Samples were first dissociated by pipetting retina and TRIzol vigorously. A 1:4 volume of chloroform (Sigma) was then added to each sample and incubated at room temperature for 3 min followed by centrifugation at 12,000 ***g*** at 4C for 15 min. The aqueous layer was then transferred to a siliconized Eppendorf tube followed by the addition of 2.0 ml glycogen (Roche) and 500 ml isopropanol (Fisher Scientific). Samples were incubated at room temperature for 10 min followed by centrifugation at 12,000 ***g*** at 4C for 15 min. Samples were then washed twice with ice-cold 80% ethanol (Thermo Fisher Scientific) to remove salts, resuspended in DEPC H_2_O and the concentration was determined with a NanoDrop (Thermo Fisher Scientific). Libraries were prepared from 500 ng total RNA with the TruSeq Stranded Total RNA Library Prep Kit according to the manufacturer's directions (Illumina). Paired-end 100-cycle sequencing was performed on HiSeq 2000 or HiSeq 2500 sequencers, according to the manufacturer's directions (Illumina).

### qRT-PCR

cDNA was made from 200 ng of RNA from H9, JHDR, 1B6, 1C9, 5D2, 8H6, 8E10, 5B2, 1C2 and 4F9 organoid lines (Applied Biosystems 4387406). cDNA was loaded onto a Custom TaqMan Array Card (Applied Biosystems 4342249) run on a QuantStudio 7 Flex (Thermo Fisher Scientific) system. ‘Undetermined’ values were set to a Ct of 40 as the limit of detection of the assay.

### RNA-seq

RNA was quantified using the Quant-iT RiboGreen assay (Life Technologies) and quality checked by 2100 Bioanalyzer RNA 6000 Nano assay (Agilent), 4200 TapeStation High Sensitivity RNA ScreenTape assay (Agilent) or LabChip RNA Pico Sensitivity assay (PerkinElmer) before library generation. Libraries were prepared from total RNA with the TruSeq Stranded Total RNA Library Prep Kit according to the manufacturer's instructions (Illumina, PN 20020599). Libraries were analyzed for insert size distribution on a 2100 BioAnalyzer High Sensitivity kit (Agilent Technologies), 4200 TapeStation D1000 ScreenTape assay (Agilent Technologies) or Caliper LabChip GX DNA High Sensitivity Reagent Kit (PerkinElmer). Libraries were quantified using the Quant-iT PicoGreen ds DNA assay (Life Technologies) or low-pass sequencing with a MiSeq nano kit (Illumina). Paired end 100 cycle sequencing was performed on a NovaSeq 6000 (Illumina). For PCA analysis, only protein-coding genes with the annotation level (https://www.gencodegenes.org/pages/data_format.html) in 1 (verified loci) and 2 (manually annotated loci) were included in the analysis. With input of read counts for all samples, counts per million mapped reads (CPMs) were obtained by using the function *cpm* in the edgeR package. Genes were removed when the corresponding CPMs for all samples were smaller than the CPM with a corresponding raw read count of 10. The top 3000 most variable genes were then selected by ranking the mean absolute deviation (MAD) of the log_2_-transformed CPMs in descending order. Based on these most variable genes, the PCA analysis was performed on the log_2_-transformed CPMs by using the *prcomp* function available in the standard R language. The top two principal components (PCs) were used to draw the PCA figures.

### HiChIP

Paired-end reads of 100 bp were trimmed for adapters cutadapt (version 1.9, paired-end mode, default parameter with ‘-m 25 -O 6’) (Martin, 2011) and then processed by HiC-Pro (version 2.11.4) (Servant et al., 2015) using mouse genome mm9 (MGSCv37 from Sanger) and Arima fragment file (Ligation sites GATC,GANTC). Bowtie2-2.2.4, samtools-1.2, R-3.4.0, Python-2.7.12 were configured for HiC-Pro. allValidPairs files from HiC-Pro pipeline were then used to generate hic file for Visualization. All our samples have good depth (209 M to 282 M pairs) and comparable metrics with previously published data (accession number GSE80820) (Mumbach et al., 2016) such as valid interaction rate (78.98% to 92.79% compared with GEO deposit 78.65% to 80.53%). Each sample called ∼20 k loops by FitHiChIP (Bhattacharyya et al., 2019) whereas only about ∼300 loops have been called as differential loops (by FitHiChIP, calling with ChIP-seq data).

### scRNA-seq

Single cell RNA-seq was performed using the 10x platform. Sequences from each individual Illumina sequencing dataset were demultiplexed using bcl2fastq v2.20.0.422 (Illumina). Sequencing reads were processed using 10x Genomics Cell Ranger version 7.0.0, with reads mapping to a custom human reference genome GRCh38_GFP_tdTomato_HAtag_LinkerHAtag-7.0.1. Quality control filtering, clustering, dimensionality reduction, visualization and differential gene expression were performed using Seurat v4.3.0.1 with R v4.2.1 (see supplementary Materials and Methods for further details).

### Vision testing

The OptoMotry system from CerebralMechanics was used to measure the optomotor response of the CRISPR gene-edited mice. Briefly, a rotating cylinder covered with a vertical sine wave grating was calculated and drawn in virtual three-dimensional (3D) space on four computer monitors facing to form a square. CRISPR gene-edited mice standing unrestrained on a platform in the center of the square tracked the grating with reflexive head and neck movements. The spatial frequency of the grating was clamped at the viewing position by repeatedly re-centering the cylinder on the head. Acuity was quantified by increasing the spatial frequency of the grating until an optomotor response could not be elicited. Contrast sensitivity was measured at spatial frequencies between 0.1 and 0.45 cyc/deg. The tester was blinded to genotype until after testing was complete. Results were plotted as mean±s.e.m.

### GFP reporter assay

0.5 μl of plasmid mixture [2 μg/μl of enhancer plasmid and 0.5 μg/μl of a pCig2-H3.3-scarlet plasmid, a normalization control generously gifted to us from the Solecki Lab (Developmental Neurobiology at St. Jude Children's Research Hospital, TN, USA), resuspended in HBSS (Corning, 21-022-CV)] was co-electroporated into the sub-retinal space of C57/BL6 mice at P0. Mouse retinae were harvested at P21 for GFP amplification immunostaining. Experiments were performed in biological triplicates for each enhancer plasmid. One 40× confocal image from three retina were scored for each construct. GFP^+^, Scarlet^+^ cell types were counted and divided by Scarlet^+^ (electroporation control) cells to calculate the percentage of GFP^+^ cells for each cell type. Cells were assigned to specific cell types based on their location and morphology. Results were plotted as mean±s.d. for each cell type for each construct.

### Organoid EdU labeling and scoring

EdU labeling was performed per manufacturer's instructions (Click-iT EdU imaging kit, Invitrogen, C10340), and DNA was stained with 0.2 μg/ml DAPI (Sigma-Aldrich). Briefly, retinal organoids in individual wells containing 1 ml of 3D-RDM were given 1 μl of EdU 1 h before fixing and cryoembedding. Organoids were then fixed by 4% PFA overnight, embedded by OCT Compound (Scigen 4583) and cryosectioned at 10 μm. Sections were fixed by 4% PFA, washed with 3% BSA-PBS, incubated with 0.5% Triton X-100 in PBS (Sigma T9284) and washed again. Cryosections were imaged using a Zeiss LSM 700 confocal microscope using a 40× lens. Three images containing at least two biological replicates were scored for each cell line. Results were plotted as mean±s.d. of manual scoring.

### Organoid caspase 3 scoring

For each genotype or human retinal organoids, three images from at least two biological replicates were collected. The fields were selected randomly using the DAPI channel to minimize bias in the caspase channel. Images were collected and then total nuclei and Caspase^+^ nuclei were scored. Nuclear fragments were not scored. The number of Caspase^+^ nuclei across the three images on a given section were combined, the total number of nuclei were combined, and the ratio and percentage were calculated. The data for the two sections were averaged and the standard deviation was calculated. The individual datapoints from independent sections were plotted together with mean±s.d.

### Imaging

Images were taken with the Zeiss LSM 700 confocal microscope using a 40× lens. Brightness and contrast were modified for images presented in the figures for the immunofluorescence studies.

### Statistics and reproducibility

Mice of both sexes were randomly selected for analyses. Cell lines were anonymized for analysis of human retinal organoids. Images were anonymized for scoring. No statistical method was used to predetermine sample size. There were no instances in which repeat experiments yielded conflicting results, suggesting reproducibility of our experiments. GraphPad Prism 8 software was used to calculate statistical measures. No data were excluded from the analyses.

## Supplementary Material

10.1242/develop.202435_sup1 Supplementary information

Table S1. Bulk RNA-seq of stage 1 human retinal organoids (FPKM).
