# Supplementary material for: Evolutionary conservation of VSX2 super-enhancer modules in retinal development
Source: Development. 2024 Jul 12;151(13):dev202435. doi: 10.1242/dev.202435 (PMC11266796; doi:10.1242/dev.202435)
Supplement: Supplementary information [file develop-151-202435-s1.pdf]

Figure S1

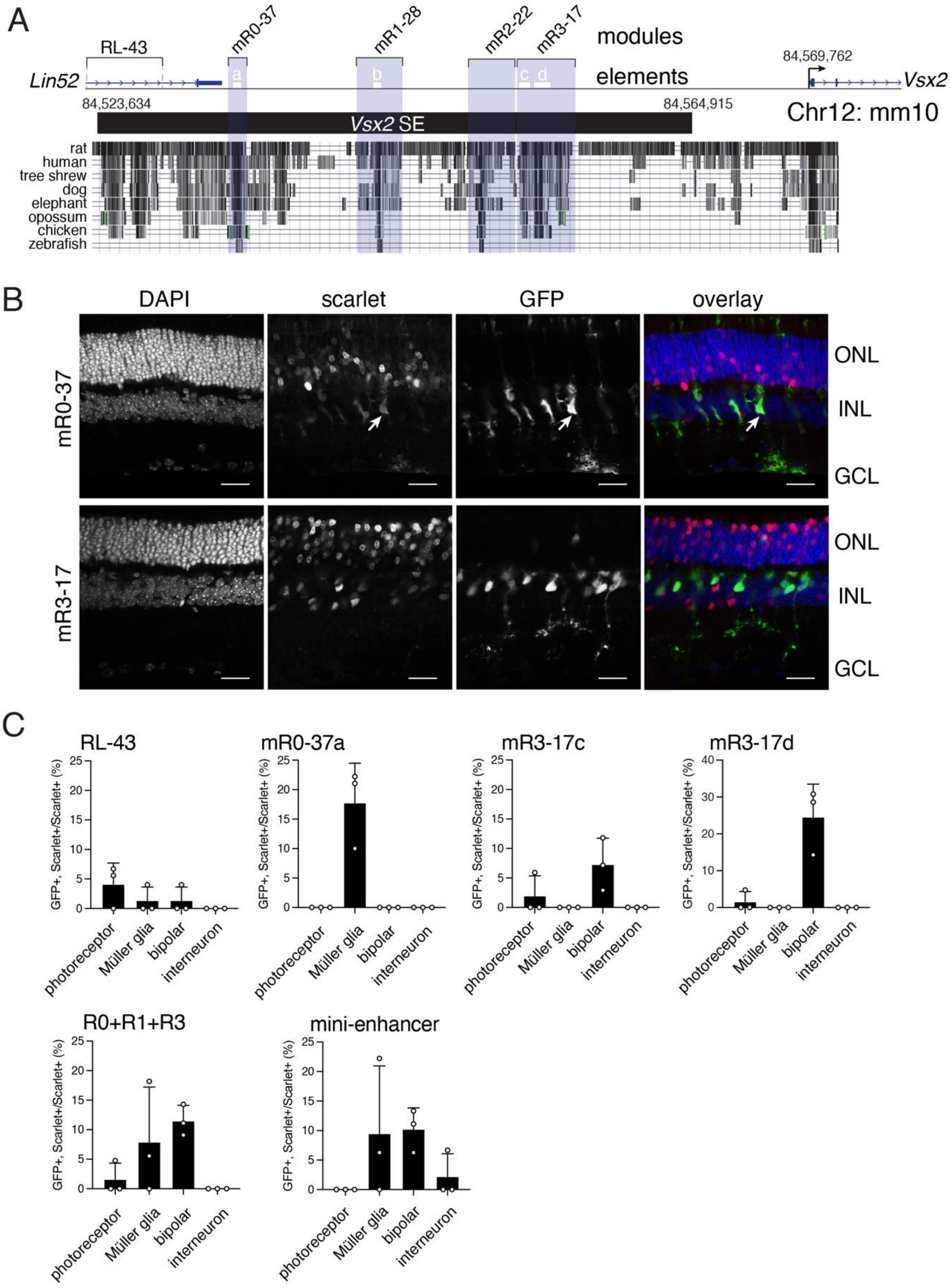

Development • Supplementary information

Fig. S1. Mouse Vsx2-SE elements drive reporter expression in a cell type-specific manner. **A)** Drawing of the original Vsx2 SE identified by H3K27Ac ChIP-seq (black bar) and the original mouse deletions with coordinates in mm10. Within these modules are additional smaller elements (a-d). **B)** Micrographs of GFP (green) and Scarlet (red) reporter expression at P21 from square wave electroporation of P0 mice. **C)** Bar plot showing mean and standard deviation of three biological replicates for each mouse reporter construct. Reporters containing mR0-37a drive expression in Müller glia and mR3-17d drives expression in bipolar neurons. Arrows in (B) indicate Müller glia for the mR0-37a element and bipolar neurons for the mR3-17d elements within the modules. Scale bar: 25 μM.

Figure 2

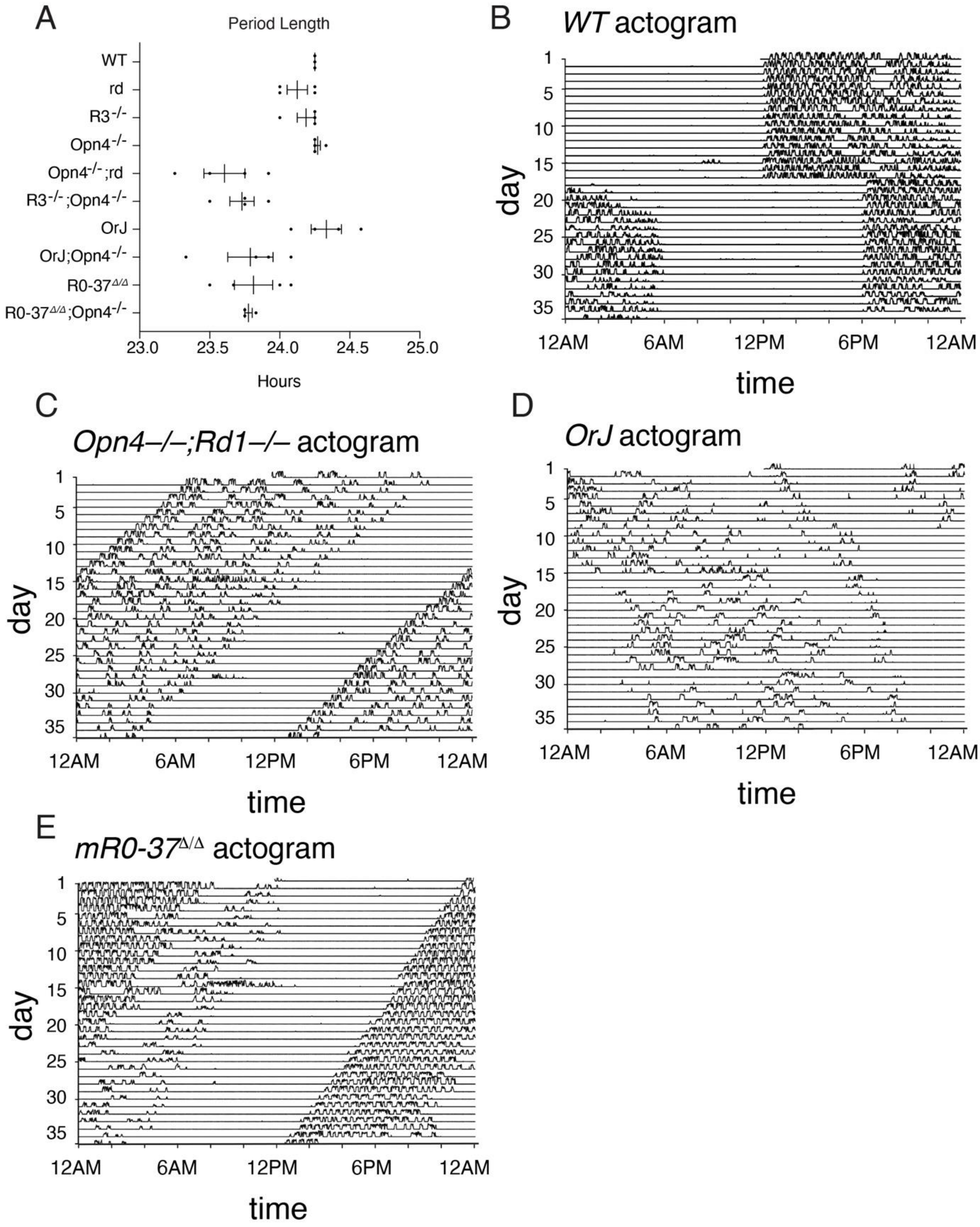

Development • Supplementary information

Fig. S2. *OrJ* and R0-37$^{\Delta/\Delta}$ mice do not photoentrain. A) Period length of the 10 mouse strains tested. Data is presented as a scatter dot plot with the line at mean with SEM. n= 38 mice total with at least three mice for each mouse strain (represented by black dots). **B-E** Representative actograms of WT, Opn4$^{-/-}$;rd, *OrJ*, and R0-37$^{\Delta/\Delta}$ adult mice over 36 days. Lights turned off at 12PM and on at 12AM days 1-17. Lights tuned off at 6PM and on at 6AM days 18-36. Running wheel activity is displayed by peak amplitude.

Table S1. Bulk RNA-seq of stage 1 human retinal organoids (FPKM).

Available for download at
https://journals.biologists.com/dev/article-lookup/doi/10.1242/dev.202435#supplementary-data

Supplementary Materials and Methods

scRNA-seq

Each dataset was initially filtered so that genes that were expressed in at least 10 or more cells and cells that expressed at least 200 genes were included. Additionally, cells with fewer than 300 genes expressed (presumed to be empty droplets or cellular debris), with fewer than 500 UMIs, more than 10% of unique transcripts derived from mitochondrial genes and more than 3 median absolute deviations (MADs) from the median number of unique transcripts derived from mitochondrial genes were removed. Afterwards, cells with more than 3 MADs from the median number of genes expressed were removed. Individual samples were then preprocessed using the standard Seurat (v4.3.0.1) workflow (NormalizeData, ScaleData, FindVariables, RunPCA, FindNeighbors, FindClusters, and RunUMAP; github.com/satijalab/Seurat). Datasets were individually log-normalized using Seurat's NormalizeData with default parameters. Cell cycle scoring was conducted using the associated S and G2M phase gene list from Tirosh et al 2015 using the CellCycleScoring command in Seurat. We calculated 3,000 features that exhibit high cell-to-cell variation in the dataset using Seurat's FindVariableFeature function. Next, we scaled the data by linear regression against the number of reads using Seurat's ScaleData function with default parameters. The variable genes were projected onto a low-dimensional subspace using principal component analysis using Seurat's RunPCA function with default parameters. The number of principal components (Npcs) were selected based on inspection of the plot of variance explained (Npcs = 30).

Datasets were integrated using Harmony with default parameters. A shared-nearest-neighbor graph was constructed based on the Euclidean distance in the low-dimensional subspace using Seurat's FindNeighbors with dims = 1:30 and default parameters. Integrated

Development • Supplementary information

datasets then underwent non-linear dimensional reduction and visualization using UMAP. Clusters were identified using a resolution of 0.3 and the Leiden algorithm for the integrated datasets.

Cell types were assigned to each cell based on their highest cell type module score created from the top fifty genes for each cell type from adult wildtype controls[1]. Furthermore, clusters at a resolution of 0.3 where then curated into cell type specific clusters using the cell type module scores as a guide.

Bipolar cells were subsetted from Seurat object of fully integrated samples, split up by their individual sample IDs, reintegrated using Harmony as described above and reclustered.

Plasmid Constructs

Mouse Region 0 was inserted into pUC57 vectors with SalI/HindIII cloning sites (synthesized by GenScript). Underlined sequences were added to the 5' and 3'.

Vsx2SE-Region0 sequence (length 983 bp):

GTCGACACTAGGTCTAACTGGCTGTTCATATCTGTTTAGACCTTTGCCACTTT…TGC AGTGAGGTTCTTACCACCATGTCAAAGAGAATTTACCTCGAGAAGCTT

The plasmid was transformed and CsCl prepped. Diagnostic digests and Sanger sequencing were used for plasmid validation. The Mouse Region 0 plasmid was cut using SalI (NEB R0138S) and XhoI (NEB R0146S) with Buffer 3.1 (NEB B7203S) and cloned into the SalI and XhoI sites of Chx10 (164bp crit reg+SV40 bas prom)-GFP-IRES-AP. Removal of the 164 bp critical region constituted the negative control plasmid. Chx10 (164bp crit reg+SV40 bas prom)-GFP-IRES-AP12 constituted the positive control plasmid. Diagnostic digests and sequencing were used for plasmid validation. Chx10 (164 bp crit reg+SV40 bas prom)-GFP-IRES-AP[2] was a

Development • Supplementary information

gift from Connie Cepko (Addgene plasmid # 18808 ; http://n2t.net/addgene:18808 ; RRID:Addgene_18808).

The Human Regions and refined mouse regions have the same plasmid design as those above and were fully synthesized by GenScript. Plasmids are deposited in Addgene. These plasmids were transformed, CsCl prepped, and validated as previously described. Underlined sequences were added to the 5' and 3' end.

Lin52 Region (RL-43) mm10 chr12:84520277-84526527 (length 6251bp):

GTCGACAACTGGAACCATTGTCAGTT…CTGCTCATTTTCCCTTTAAACTCGAGAAGCTT

R0-a mm9 chr12:85873175-85873675 (length 501 bp):

GTCGACCTGGAGTGCTGGCAGAGCCT….GAGGGATGGGGAACACGGTCCTCGAGAAGCTT

R1-b mm9 chr12:85882488-85882769 (length 282 bp):

GTCGACGATCTGCCCAGCCCTCCCCT….TTAGGATCAGTGCTGTGCACCTCGAGAAGCTT

R3-c mm9 chr12:85892001-85892193 (length 193 bp):

GTCGACGGGGGTAGGGGAAGCTCCCA….GAGGGACAAGAGAAAGAAACCTCGAGAAGCTT

R3-d mm9 chr12:85892675-85893325 (length 651 bp):

GTCGACTGTGGGTACTCAGGGTCACA….TACACGAATTAAAACGGCTCCTCGAGAAGCTT

R0 Hg19 chr14:74670103-74671150 (length 1048 bp):

GTCGACATGGACTGGAACTGATTTTT….CCATTGCAAAGAAAGCTAACCTCGAGAAGCTT

R1 Hg19 chr14:74679525-74682829 (length 3305 bp):

GTCGACGGAGGGGAGGAAGGAAATAG….TGTTATGTTTAATGAATTATCTCGAGAAGCTT

R2 Hg19 chr14:74684703-74687605 (length 2903 bp):

GTCGACAGGCCGACCCTATGCTCCCT….GGATTTTGTGAGAATTAAATCTCGAGAAGCTT

R3 Hg19 chr14:74687802-74692133 (length 4332 bp):

GTCGACCTCACAGCTCATGACATGCC….TCCTCTAGGAAGTTTTCCCTCTCGAGAAGCTT

Development • Supplementary information

Vector builds for mouse Regions 1, 2, 3, consensus, and positive control are reported in the Supplementary Information of Honnell et al., 2022[1].

References

1 Honnell, V. *et al.* Identification of a modular super-enhancer in murine retinal development. *Nature Communications* **13**, 253, doi:10.1038/s41467-021-27924-y (2022).

2 Kim, D. S., Matsuda, T. & Cepko, C. L. A core paired-type and POU homeodomain-containing transcription factor program drives retinal bipolar cell gene expression. *The Journal of neuroscience : the official journal of the Society for Neuroscience* **28**, 7748-7764, doi:28/31/7748 [pii]

10.1523/JNEUROSCI.0397-08.2008 (2008).

Development • Supplementary information
